# Supplementary material for: Role and mechanism of NCAPD3 in promoting malignant behaviors in gastric cancer
Source: Front Pharmacol. 2024 Apr 22;15:1341039. doi: 10.3389/fphar.2024.1341039 (PMC11070777; doi:10.3389/fphar.2024.1341039)
Supplement: Supplementary file 11 [file DataSheet2.ZIP › GSEA/GO_CC/my_analysis.Gsea.1599461408869/gsea_report_for_WT_1599461408869.html]

Report for WT 1599461408869 [GSEA]

| GS  follow link to MSigDB | GS DETAILS | SIZE | ES | NES | NOM p-val | FDR q-val | FWER p-val | RANK AT MAX | LEADING EDGE || 1 | GO\_RIBONUCLEOPROTEIN\_COMPLEX | Details ... | 76 | 0.35 | 2.29 | 0.000 | 0.007 | 0.009 | 848 | tags=93%, list=60%, signal=222% |
| 2 | GO\_INTRINSIC\_COMPONENT\_OF\_PLASMA\_MEMBRANE | Details ... | 110 | 0.29 | 2.05 | 0.000 | 0.031 | 0.075 | 170 | tags=31%, list=12%, signal=32% |
| 3 | GO\_PRERIBOSOME | Details ... | 25 | 0.38 | 1.70 | 0.032 | 0.340 | 0.723 | 837 | tags=96%, list=59%, signal=232% |
| 4 | GO\_MITOCHONDRION | Details ... | 149 | 0.20 | 1.54 | 0.021 | 0.664 | 0.958 | 588 | tags=53%, list=42%, signal=81% |
| 5 | GO\_SITE\_OF\_POLARIZED\_GROWTH | Details ... | 20 | 0.36 | 1.50 | 0.072 | 0.643 | 0.979 | 183 | tags=35%, list=13%, signal=40% |
| 6 | GO\_METHYLTRANSFERASE\_COMPLEX | Details ... | 18 | 0.36 | 1.46 | 0.090 | 0.660 | 0.990 | 847 | tags=94%, list=60%, signal=233% |
| 7 | GO\_MITOCHONDRIAL\_PROTEIN\_COMPLEX | Details ... | 17 | 0.36 | 1.43 | 0.104 | 0.685 | 0.997 | 838 | tags=94%, list=59%, signal=229% |
| 8 | GO\_MITOCHONDRIAL\_MATRIX | Details ... | 46 | 0.25 | 1.41 | 0.086 | 0.638 | 0.997 | 585 | tags=57%, list=41%, signal=93% |
| 9 | GO\_RIBOSOME | Details ... | 15 | 0.37 | 1.41 | 0.119 | 0.573 | 0.997 | 892 | tags=100%, list=63%, signal=269% |
| 10 | GO\_SPLICEOSOMAL\_COMPLEX | Details ... | 18 | 0.34 | 1.40 | 0.104 | 0.544 | 0.999 | 848 | tags=94%, list=60%, signal=234% |
| 11 | GO\_LATE\_ENDOSOME | Details ... | 19 | 0.33 | 1.36 | 0.118 | 0.601 | 1.000 | 373 | tags=53%, list=26%, signal=71% |
| 12 | GO\_CATALYTIC\_COMPLEX | Details ... | 135 | 0.19 | 1.36 | 0.087 | 0.560 | 1.000 | 513 | tags=45%, list=36%, signal=64% |
| 13 | GO\_SOMATODENDRITIC\_COMPARTMENT | Details ... | 56 | 0.23 | 1.35 | 0.116 | 0.540 | 1.000 | 325 | tags=36%, list=23%, signal=45% |
| 14 | GO\_LAMELLIPODIUM | Details ... | 15 | 0.34 | 1.28 | 0.191 | 0.684 | 1.000 | 339 | tags=47%, list=24%, signal=61% |
| 15 | GO\_TRANSFERASE\_COMPLEX | Details ... | 81 | 0.19 | 1.25 | 0.175 | 0.721 | 1.000 | 503 | tags=47%, list=36%, signal=69% |
| 16 | GO\_NEURON\_PROJECTION | Details ... | 92 | 0.17 | 1.18 | 0.227 | 0.863 | 1.000 | 250 | tags=27%, list=18%, signal=31% |
| 17 | GO\_DISTAL\_AXON | Details ... | 25 | 0.25 | 1.15 | 0.294 | 0.938 | 1.000 | 183 | tags=28%, list=13%, signal=32% |
| 18 | GO\_APICAL\_PLASMA\_MEMBRANE | Details ... | 26 | 0.25 | 1.15 | 0.281 | 0.886 | 1.000 | 411 | tags=42%, list=29%, signal=59% |
| 19 | GO\_APICAL\_JUNCTION\_COMPLEX | Details ... | 18 | 0.28 | 1.12 | 0.333 | 0.907 | 1.000 | 99 | tags=22%, list=7%, signal=24% |
| 20 | GO\_TIGHT\_JUNCTION | Details ... | 17 | 0.28 | 1.11 | 0.325 | 0.886 | 1.000 | 99 | tags=24%, list=7%, signal=25% |
| 21 | GO\_CHROMOSOMAL\_REGION |  | 24 | 0.25 | 1.11 | 0.330 | 0.846 | 1.000 | 11 | tags=13%, list=1%, signal=12% |
| 22 | GO\_DENDRITIC\_TREE |  | 38 | 0.21 | 1.10 | 0.355 | 0.849 | 1.000 | 244 | tags=29%, list=17%, signal=34% |
| 23 | GO\_APICAL\_PART\_OF\_CELL |  | 32 | 0.21 | 1.07 | 0.364 | 0.912 | 1.000 | 428 | tags=44%, list=30%, signal=61% |
| 24 | GO\_RIBONUCLEOPROTEIN\_GRANULE |  | 24 | 0.23 | 1.06 | 0.391 | 0.887 | 1.000 | 244 | tags=29%, list=17%, signal=35% |
| 25 | GO\_RNA\_POLYMERASE\_II\_TRANSCRIPTION\_FACTOR\_COMPLEX |  | 18 | 0.25 | 1.03 | 0.411 | 0.939 | 1.000 | 132 | tags=22%, list=9%, signal=24% |
| 26 | GO\_CELL\_BODY |  | 40 | 0.19 | 1.03 | 0.418 | 0.906 | 1.000 | 325 | tags=33%, list=23%, signal=41% |
| 27 | GO\_FIBRILLAR\_CENTER |  | 19 | 0.24 | 1.01 | 0.425 | 0.918 | 1.000 | 877 | tags=89%, list=62%, signal=233% |
| 28 | GO\_CENTROSOME |  | 52 | 0.17 | 0.99 | 0.448 | 0.935 | 1.000 | 424 | tags=38%, list=30%, signal=53% |
| 29 | GO\_NUCLEAR\_TRANSCRIPTION\_FACTOR\_COMPLEX |  | 20 | 0.22 | 0.95 | 0.533 | 1.000 | 1.000 | 132 | tags=20%, list=9%, signal=22% |
| 30 | GO\_CULLIN\_RING\_UBIQUITIN\_LIGASE\_COMPLEX |  | 19 | 0.22 | 0.94 | 0.543 | 1.000 | 1.000 | 338 | tags=37%, list=24%, signal=48% |
| 31 | GO\_NUCLEOLUS |  | 127 | 0.13 | 0.94 | 0.545 | 0.988 | 1.000 | 848 | tags=74%, list=60%, signal=169% |
| 32 | GO\_NUCLEAR\_BODY |  | 88 | 0.14 | 0.92 | 0.566 | 0.987 | 1.000 | 243 | tags=22%, list=17%, signal=24% |
| 33 | GO\_EARLY\_ENDOSOME\_MEMBRANE |  | 18 | 0.22 | 0.92 | 0.554 | 0.958 | 1.000 | 373 | tags=39%, list=26%, signal=52% |
| 34 | GO\_TRANSCRIPTION\_FACTOR\_COMPLEX |  | 27 | 0.19 | 0.89 | 0.582 | 1.000 | 1.000 | 132 | tags=19%, list=9%, signal=20% |
| 35 | GO\_ORGANELLE\_INNER\_MEMBRANE |  | 40 | 0.16 | 0.87 | 0.632 | 1.000 | 1.000 | 729 | tags=65%, list=52%, signal=131% |
| 36 | GO\_ENVELOPE |  | 104 | 0.12 | 0.86 | 0.684 | 1.000 | 1.000 | 660 | tags=53%, list=47%, signal=92% |
| 37 | GO\_MITOCHONDRIAL\_ENVELOPE |  | 60 | 0.14 | 0.86 | 0.665 | 0.981 | 1.000 | 84 | tags=12%, list=6%, signal=12% |
| 38 | GO\_NEURON\_TO\_NEURON\_SYNAPSE |  | 25 | 0.18 | 0.83 | 0.674 | 1.000 | 1.000 | 317 | tags=32%, list=22%, signal=41% |
| 39 | GO\_TRANSFERASE\_COMPLEX\_TRANSFERRING\_PHOSPHORUS\_CONTAINING\_GROUPS |  | 21 | 0.19 | 0.82 | 0.676 | 1.000 | 1.000 | 83 | tags=14%, list=6%, signal=15% |
| 40 | GO\_OUTER\_MEMBRANE |  | 21 | 0.19 | 0.82 | 0.705 | 0.997 | 1.000 | 744 | tags=71%, list=53%, signal=149% |
| 41 | GO\_NUCLEAR\_SPECK |  | 46 | 0.15 | 0.81 | 0.718 | 0.982 | 1.000 | 489 | tags=41%, list=35%, signal=61% |
| 42 | GO\_POSTSYNAPSE |  | 38 | 0.15 | 0.81 | 0.715 | 0.959 | 1.000 | 317 | tags=29%, list=22%, signal=36% |
| 43 | GO\_MICROTUBULE\_ORGANIZING\_CENTER |  | 66 | 0.13 | 0.80 | 0.758 | 0.957 | 1.000 | 73 | tags=11%, list=5%, signal=11% |
| 44 | GO\_CILIUM |  | 34 | 0.15 | 0.77 | 0.788 | 0.982 | 1.000 | 60 | tags=12%, list=4%, signal=12% |
| 45 | GO\_CELL\_LEADING\_EDGE |  | 39 | 0.14 | 0.76 | 0.775 | 0.981 | 1.000 | 428 | tags=36%, list=30%, signal=50% |
| 46 | GO\_PLASMA\_MEMBRANE\_PROTEIN\_COMPLEX |  | 24 | 0.17 | 0.75 | 0.773 | 0.966 | 1.000 | 281 | tags=25%, list=20%, signal=31% |
| 47 | GO\_ENDOSOME\_MEMBRANE |  | 41 | 0.14 | 0.74 | 0.783 | 0.962 | 1.000 | 373 | tags=32%, list=26%, signal=42% |
| 48 | GO\_MICROTUBULE\_CYTOSKELETON |  | 99 | 0.11 | 0.74 | 0.855 | 0.946 | 1.000 | 73 | tags=9%, list=5%, signal=9% |
| 49 | GO\_CHROMOSOME |  | 136 | 0.10 | 0.73 | 0.861 | 0.937 | 1.000 | 89 | tags=9%, list=6%, signal=9% |
| 50 | GO\_NUCLEAR\_PERIPHERY |  | 17 | 0.17 | 0.69 | 0.843 | 0.964 | 1.000 | 862 | tags=82%, list=61%, signal=209% |
| 51 | GO\_NUCLEAR\_ENVELOPE |  | 47 | 0.12 | 0.68 | 0.875 | 0.954 | 1.000 | 970 | tags=85%, list=69%, signal=263% |
| 52 | GO\_UBIQUITIN\_LIGASE\_COMPLEX |  | 32 | 0.13 | 0.66 | 0.881 | 0.957 | 1.000 | 503 | tags=44%, list=36%, signal=66% |
| 53 | GO\_WHOLE\_MEMBRANE |  | 127 | 0.09 | 0.64 | 0.957 | 0.956 | 1.000 | 383 | tags=28%, list=27%, signal=34% |
| 54 | GO\_VACUOLAR\_MEMBRANE |  | 41 | 0.10 | 0.57 | 0.948 | 0.987 | 1.000 | 366 | tags=29%, list=26%, signal=38% |
| 55 | GO\_RUFFLE |  | 17 | 0.14 | 0.56 | 0.929 | 0.974 | 1.000 | 1213 | tags=100%, list=86%, signal=704% |
| 56 | GO\_NUCLEAR\_MEMBRANE |  | 26 | 0.10 | 0.49 | 0.978 | 0.985 | 1.000 | 1005 | tags=88%, list=71%, signal=302% |
Table: Gene sets enriched in phenotype **WT (3 samples) **[plain text format]****
